# Melatonin suppresses chronic restraint stress-mediated metastasis of epithelial ovarian cancer via NE/AKT/β-catenin/SLUG axis

**DOI:** 10.1038/s41419-020-02906-y

**Published:** 2020-08-18

**Authors:** Shixia Bu, Qian Wang, Junyan Sun, Xiao Li, Tingting Gu, Dongmei Lai

**Affiliations:** 1 The International Peace Maternity and Child Health Hospital, School of Medicine, Shanghai Jiaotong University, Shanghai, 200030 China; 2 Shanghai Key Laboratory of Embryo Original Disease, School of Medicine, Shanghai Jiaotong University, Shanghai, 200030 China; 3 Department of Obstetrics and Gynaecology, Shanghai Sixth People’s Hospital, Shanghai Jiaotong University, Shanghai, 200233 China

**Keywords:** Ovarian cancer, Pharmaceutics

## Abstract

Chronic stress has been shown to facilitate progression of epithelial ovarian cancer (EOC), however, the neuro-endocranial mechanism participating in this process still remains unclear. Here, we reported that chronic restraint stress (CRS) promoted the abdominal implantation metastasis of EOC cells and the expression of epithelial–mesenchymal transition-related markers in tumor-bearing mouse model, including TWIST, SLUG, SNAIL, and β-catenin. We observed that β-catenin co-expressed with SLUG and norepinephrine (NE) in tumor tissues obtained from nude mice. Further ex vivo experiments revealed that NE promoted migration and invasion of ovarian cancer cells and SLUG expression through upregulating expression and improving transcriptional function of β-catenin in vitro. A human phosphor-kinase array suggested that NE activated various kinases in ovarian cancer cells, and we further confirmed that AKT inhibitor reduced NE-mediated pro-metastatic impacts and activation of the β-catenin/SLUG axis. Furthermore, the expression levels of NE and β-catenin were examined in ovarian tumor tissues by using tumor tissue arrays. Results showed that the expression levels of both NE and β-catenin were associated with poor clinical stage of serous EOC. Moreover, we found that melatonin (MLT) effectively reduced the abdominal tumor burden of ovarian cancer induced by CRS, which was partially related to the inhibition of the NE/AKT/β-catenin/SLUG axis. Collectively, these findings suggest a novel mechanism for CRS-mediated ovarian cancer metastasis and MLT has a potential therapeutic efficacy against ovarian cancer.

## Introduction

Epithelial ovarian cancer (EOC) is the most lethal gynecological cancer worldwide. Although various forms of therapy have been created, more than 70% of patients will relapse within 18 months^1^. Depression is reported to be related to a modestly risk of ovarian cancer^2^ and shorter survival in patients with ovarian cancer^3^. As is known to all, cancer patients usually live under chronic mental stress due to diagnosis-associated strong emotions, depression, and inquietude. However, whether and how neuro-endocrine regulatory networks affect biological behaviors of cancer cells still remains unclear.

Psychological stress is a common modern health problem that significantly affects our daily lives and health, which can be classified into acute stress and chronic stress. Catecholamines are hormones released during a stress response, especially by chronic stress, including norepinephrine (NE) and epinephrine (Epi)^4^. Stress-related NE and Epi are considered to worsen progression and prognosis of ovarian cancer through promoting angiogenesis^5,6^, aggressiveness^7^, chemo-resistance^8^. NE and Epi are widely reported to facilitate ovarian cancer progression via binding to β2 adrenergic receptor, which results in activation of cyclic AMP/Protein Kinase A axis and phosphorylation of several pro-tumoral proteins (reviewed in^9^). In patients with ovarian cancer, Julie et al. found a positive correlation among brain-derived neurotrophic factor (BDNF), nerve counts, and intertumoral NE content, which was significantly associated with advanced tumor stage, presence of ascites, and worse prognosis^10^. These previous studies shed light on the mechanisms that neuro-endocrine stress hormones might regulate key cellular and molecular process in cancer progression.

Epithelial–mesenchymal transition (EMT) plays a pivotal role in metastatic cascade of cancer cells. Many studies demonstrated that catecholamine hormones induced EMT and promoted metastatic capacity in various kinds of cancer, including pancreatic cancer^11^, gastric cancer^12^, ovarian cancer^7^, etc. EMT is complexly orchestrated by numerous EMT-inducing transcription factors, such as SNAIL, SLUG, TWIST, β-catenin, etc. Choi et al.^7^ reported that NE facilitated ovarian cancer aggressiveness through hTERT-mediated SLUG expression. However, limited researches reported the effects of stress-related hormones on ovarian cancer. To extend our knowledge about NE-induced ovarian cancer progression and figure out the molecular mechanisms behind it, we investigated the influences of chronic restraint stress (CRS) on ovarian cancer metastasis in nude mice models and NE-mediated regulation of EMT-related molecules in cancer cells in vitro.

Melatonin (MLT) is an endogenously-synthesized lipid soluble hormone whose level changes with circadian rhythm. MLT is considered as a potential agent to treat ovarian cancer because of its anti-proliferation, antioxidant, antiangiogenesis, and immunoregulation effects on ovarian cancer (reviewed in^13^). Zhao et al.^14^ observed a significant deficiency of serum MLT in women with ovarian cancer compared with healthy women, which suggested additional supplement of MLT might be beneficial for patients with ovarian cancer. Moreover, MLT was found to ameliorate chronic stress-induced depression-like behaviors^15^ and regulate gene expression of catecholamine biosynthesizing enzymes^16^ in animal models. Therefore, the goal of this study is to reveal the mechanism of catecholamine-mediated pro-metastatic effects on ovarian cancer and evaluate whether MLT has a potential anticancer effects in stressed nude mice bearing ovarian cancer.

## Results

### CRS facilitates metastasis of epithelial ovarian cancer in nude mouse models

**Fig. 1:**
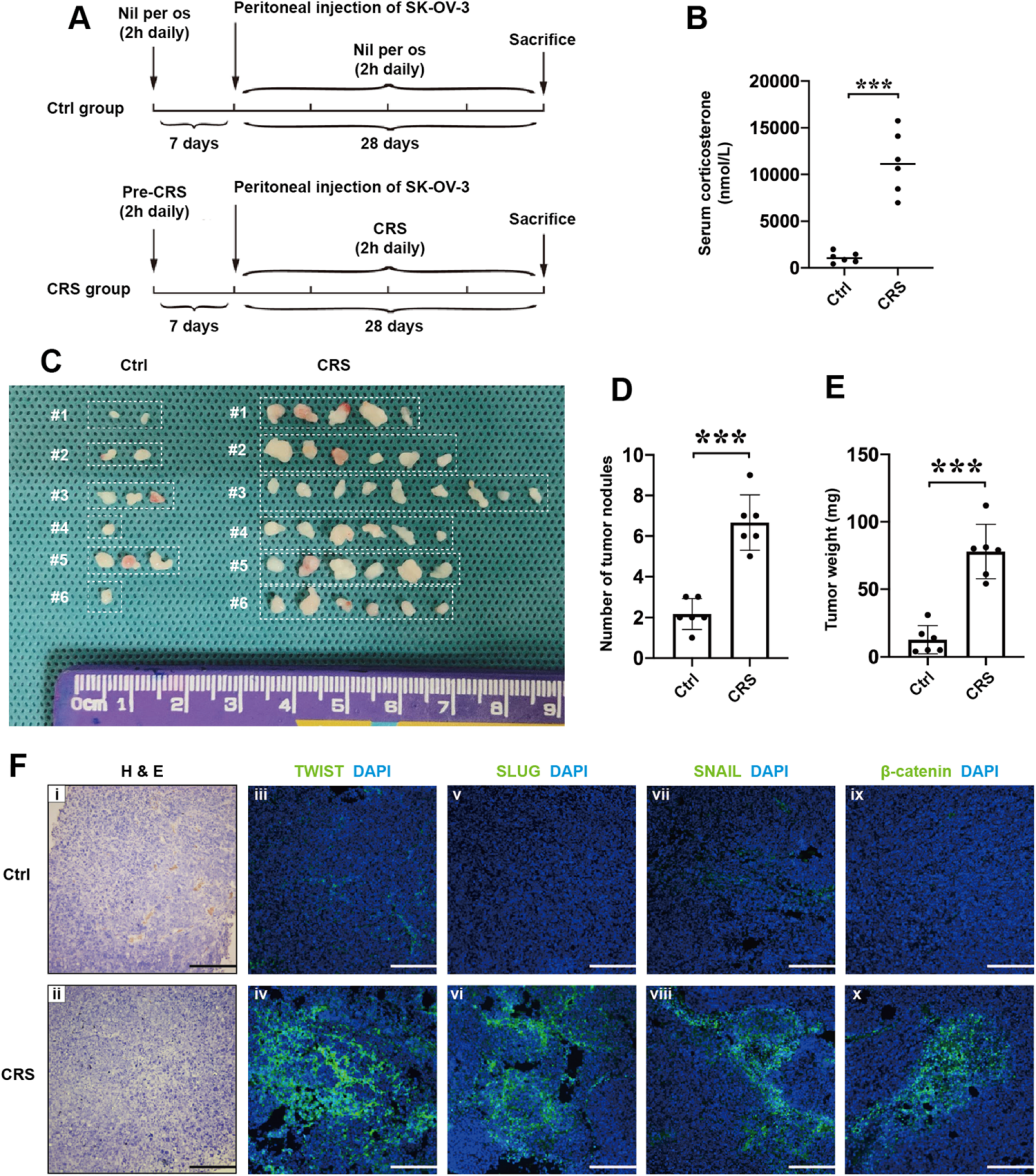
CRS promotes metastasis of SK-OV-3 ovarian cancer cells in nude mouse models. **a** The schematic graph of establishing chronic restraint stress (CRS) animal models in this study. **b** ELISA was used to evaluate the effects of CRS on nude mice by examining the concentration of serum corticosterone (*n* = 6). **c** Gross morphology of the abdominally implanted tumor burden of ovarian cancer cells in nude mice. Quantification of abdominal tumor nodules (**d**) and tumor weights (**e**) of the control group and the CRS group (*n* = 6). **f** Representative images of H&E staining (i, ii), IF staining detecting EMT-related transcription factors, including TWIST (iii, iv), SLUG (v, vi), SNAIL (vii, viii), and β-catenin (ix, x) in tumor tissues obtained from nude mouse models. *P < 0.05; ***P < 0.001. Scale bar, 100 μM.

In this study, we stressed nude mice with a physical restraint strategy based on what we previously described^17^. Before SK-OV-3 cells were intraperitoneally inoculated, we gave them 2 h of daily immobilization for a week to let nude mice stay in the state of chronic stress. After being injected 2 × 10^6^ SK-OV-3 cells intraperitoneally, CRS nude mice were received daily immobilization for another 28 days and mice in the control group were deprived of food and water at the same time (Fig. 1a). The concentration of serum corticosterone, one of chronic stress-related hormones, was higher in the nude mice of CRS group compared with control group (Fig. 1b), which indicated our models were efficiently established and induced changes of hormone secretion. We then observed that the number of abdominal tumor nodules increased in CRS group (Fig. 1c, d) and the tumor weight was significantly greater than that in control group (Fig. 1e). EMT-related transcription factors play important roles in cancer dissemination. Previous studies indicate that many of these factors, including TWIST^18^, SLUG^19^, and β-catenin^20^, act synergistically and reciprocally with one another and use common pathways (reviewed in^21^), which are associated with poor prognosis of EOC. Then we conducted immunofluorescence (IF) staining to examine whether CRS affected expression of these factors in tumor tissues. The H&E staining showed that the tumor tissues obtained from control and CRS groups were characterized as serous adenocarcinoma. IF staining revealed the increasing expression levels of TWIST, SLUG, SNAIL, and β-catenin in CRS groups compared with that in control group (Fig. 1f).

### CRS activates the β-catenin/SLUG axis in nude mouse models and in vitro

**Fig. 2:**
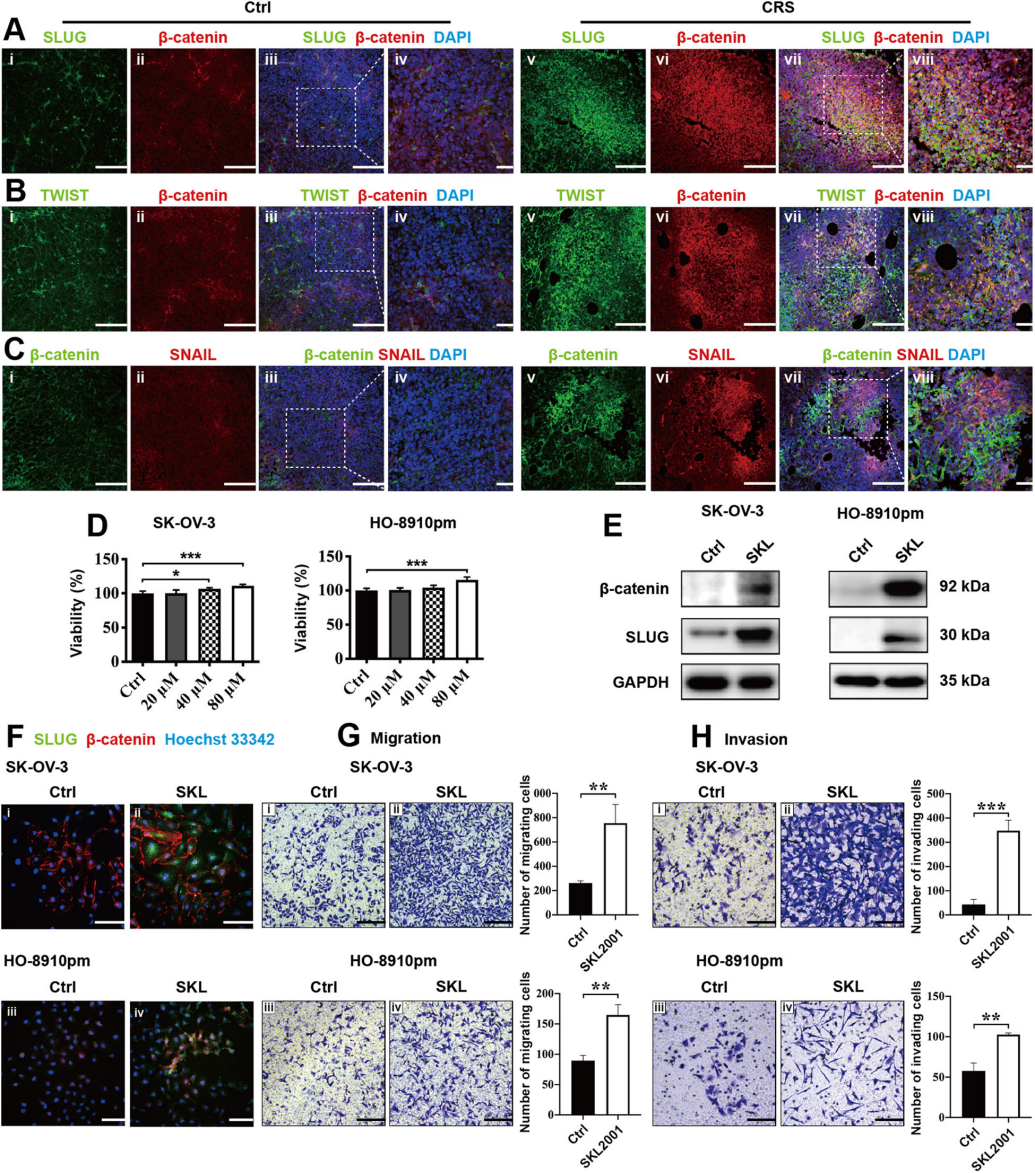
CRS activates the β-catenin/SLUG axis in nude mouse models and in vitro. Co-expression of β-catenin/SLUG (**a**), β-catenin/TWIST (**b**), and β-catenin/SNAIL (**c**) in the tumor tissues obtained from nude mouse models was evaluated by confocal laser scanning microscopy. **d** CCK-8 assay was used to test the effects of β-catenin agonist SKL2001 in different concentration on the viability of ovarian cancer cells at 24 h. Western blot (**e**) and double immunofluorescence staining (**f**) were used to examine the impacts of SKL2001 on the expression of β-catenin and SLUG in ovarian cancer cells at 24 h. Transwell assays were used to test the effects of SKL2001 on the migration (**g**) and invasion (**h**) ability of ovarian cancer cells in vitro at 24 h. *P < 0.05; **P < 0.01; ***P < 0.001. Scale bar, 200 μM.

It is widely reported the correlation between WNT/GSK-3β/β-catenin axis and EMT-related molecules, such as SNAIL^22^, SLUG^23^, and TWIST^24^. In this study, we conducted double IF staining to examine whether CRS promoted co-expression of β-catenin and EMT markers in tumor tissues. We observed that the expression levels of TWIST, SLUG, SNAIL, and β-catenin significantly increased in the CRS group. In comparison with the rare co-expression signals found in control group, we found more yellow fluorescence signals within tumor tissues from CRS group. Moreover, the distribution pattern of SLUG (green) was similar with that of β-catenin (red) and more yellow fluorescence signals were observed (Fig. 2a) than that within tissues stained with TWIST (green)/β-catenin (red) (Fig. 2b) or SNAIL (red)/β-catenin (green) from the CRS group (Fig. 2c). These morphological results suggest that CRS promotes expression of EMT-related molecules and β-catenin might directly regulate SLUG expression rather than SNAIL and TWIST in the CRS nude mouse models, which needs to be further studied.

To further evaluate whether β-catenin regulates SLUG expression, a β-catenin agonist (SKL2001, SKL) was used. First, we conducted Cell Counting Kit-8 (CCK-8) viability assay to determine a suitable work concentration of SKL to avoid nonspecific toxicity on ovarian cancer cells. According to the results of CCK-8 assay, SKL slightly increased the viability of EOC cells in vitro and we chose 20 μM as the work concentration of SKL used in subsequent experiments (Fig. 2d). Western blotting and double IF staining detecting β-catenin (red)/SLUG (green) were conducted to evaluate the effects of SKL on the expression levels of β-catenin and SLUG. We found that SKL increased the expression of β-catenin and SLUG both in SK-OV-3 and HO-8910pm cells by western blotting (Fig. 2e) and IF staining (Fig. 2f). Using Transwell migration (Fig. 2g) and invasion (Fig. 2h) assays, we observed that SKL significantly improved migration and invasion ability of both SK-OV-3 and HO-8910pm cells in vitro. These results suggest that activation of β-catenin in EOC cells promotes the expression of SLUG and increases the motility of EOC cells in vitro.

### CRS-related NE promotes the motility of EOC cells through the β-catenin/SLUG axis in nude mouse models and in vitro

**Fig. 3:**
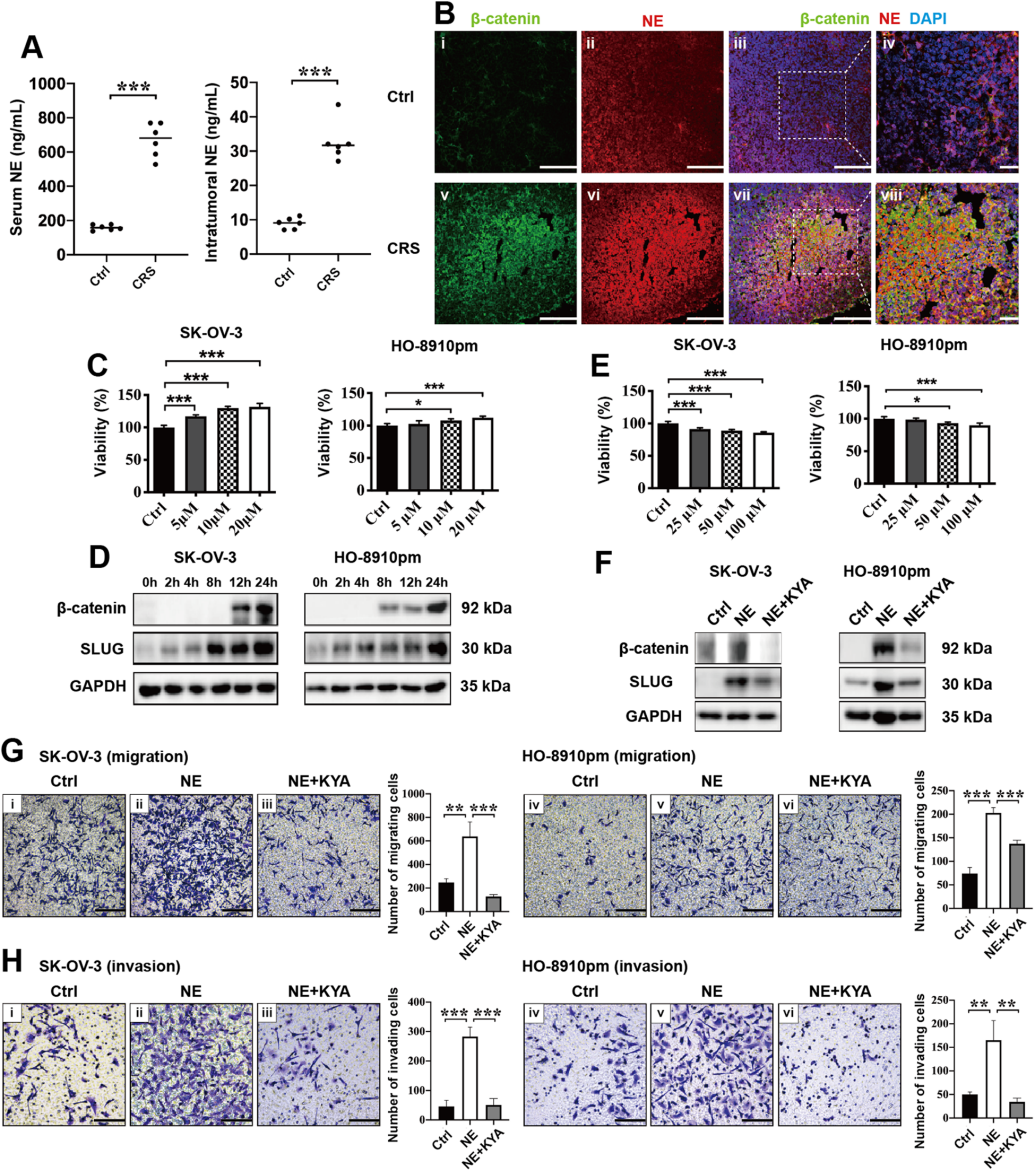
CRS-related NE promotes the motility of epithelial ovarian cancers via the β-catenin/SLUG axis in nude mouse models and in vitro. **a** Content of NE in mouse serum and tumor tissues from the control group and the CRS group was evaluated by ELISA (*n* = 6). **b** Co-expression of β-catenin/NE in the tumor tissues obtained from animal models was examined by confocal laser scanning microscopy. CCK-8 assay was used to test the effects of different concentration of NE (**c**) and β-catenin inhibitor KYA1797K (**e**) on the viability of ovarian cancer cells at 24 h. **d** NE increased the expression of β-catenin and SLUG in ovarian cancer cells time-dependently. **f** Results from western blot showed that KYA inhibited NE-mediated upregulation of β-catenin and SLUG in ovarian cancer cells at 24 h. KYA reduced NE-induced pro-migration (**g**) and pro-invasion (**h**) effects on ovarian cancer cells at 24 h. *P < 0.05; **P < 0.01; ***P < 0.001. Scale bar, 200 μM.

Emerging evidences reveal that chronic stress can promote ovarian cancer progression through neuro-endocrine-regulatory networks, such as through NE and Epi^25^. NE was found to activate WNT/β-catenin signaling in cardiomyocytes in vitro^26^ and promote SLUG expression in EOC cells in vitro^7^. Whether NE influences the β-catenin/SLUG axis still remains unknown in ovarian cancer. First, the concentration of NE in serum and tumor tissue lysates was examined. We observed that the concentration of serum NE was higher than that of intratumoral NE. In addition, concentration of serum NE and intratumoral NE in the CRS groups was higher than that in the control groups (Fig. 3a). Then we used double IF staining to test the co-expression of NE and β-catenin in tumor tissues. Results showed that the levels of both NE and β-catenin elevated in the tumor tissues from CRS group and NE co-expressed with β-catenin as similar as that of SLUG/β-catenin (Fig. 3b). CCK-8 assay showed that NE promoted the viability of EOC cells dose-dependently in vitro (Fig. 3c). Results from western blotting showed that NE promoted the expression of β-catenin and SLUG in both SK-OV-3 and HO-8910pm cells time-dependently in vitro (Fig. 3d). To investigate whether NE increases SLUG expression and motility of EOC cells via β-catenin, we downregulated the expression of β-catenin pharmacologically by a β-catenin inhibitor KYA1797K (KYA). We found that KYA inhibited cellular proliferation (Fig. 3e) and reduced NE-induced upregulation of both β-catenin expression and SLUG expression in EOC cells in vitro (Fig. 3f). In addition, 25 μM KYA significantly inhibited NE-mediated pro-migrating (Fig. 3g) and pro-invading (Fig. 3h) effects on EOC cells in vitro. Collectively, these data reveal that CRS-related NE promotes the motility of EOC cells through the β-catenin/SLUG axis.

### NE exerts upregulating effects on phosphorylation of various phosphor-kinases in SK-OV-3 ovarian cancer cells

**Fig. 4:**
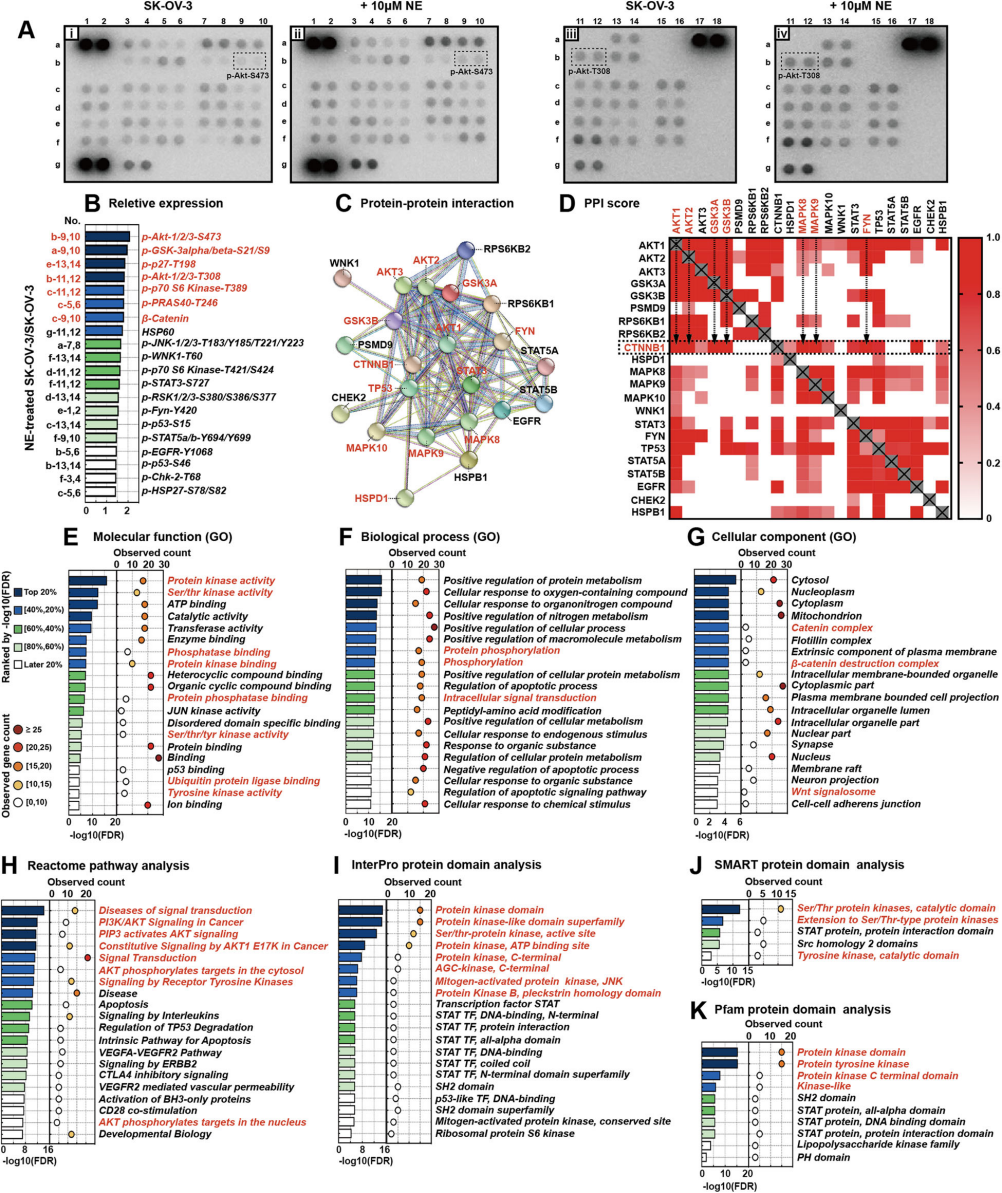
NE exerts upregulating effects on phosphorylation of various kinases in SK-OV-3 ovarian cancer cells. **a** A human phosphor-kinase array was performed to evaluate the effects of NE on the phosphorylation level of kinases in SK-OV-3 ovarian cancer cells at 24 h. **b** The list of NE-increased top 20 kinases in SK-OV-3 ranked by the relative expression. The protein–protein interaction among top 20 upregulated kinases was shown by PPI network (**c**) and PPI score (**d**). GO enrichment analysis of these kinases based on following aspects: molecular function (**e**), biological process (**f**), cellular component (**g**), and Reactome pathway analysis (**h**). NE-enhanced protein domains were analyzed by InterPro (**i**), SMART (**j**), and Pfam database (**k**).

β-catenin is a crucial component of the WNT signaling pathway. Its protein stability and degradation are complexly orchestrated by ubiquitination, phosphorylation, or other kinds of regulatory mechanisms. To elucidate whether NE regulates the β-catenin/SLUG axis and cancer motility via phosphor-kinase-mediated stabilization of β-catenin, we performed a human phosphor-kinase array to discover how NE affects the expression profile of pro-tumoral kinases in SK-OV-3 cells. Intriguingly, NE upregulated the expression of all sites in this array by more than 50% (Fig. 4a), including β-catenin (site c-9, 10). The top 20 NE-promoted phosphorylation sites and proteins are ranked by their relative expression (Fig. 4b). Among them, the top 5 increased phosphorylation residues are as following: AKT-Ser473 (2.1-fold), GSK-3α/β-Ser9/21 (2.0-fold), p27-Thr198 (1.9-fold), AKT-Thr308 (1.9-fold), p70S6K-Thr389 (1.8-fold), and PRAS40-Thr246 (1.8-fold).

Next, we used several bioinformatic databases to perform the protein–protein interaction (PPI), Gene Ontology (GO), pathway, protein domain analysis based on the top 20 proteins upregulated by NE in SK-OV-3 cells. The network graphics of PPI and the PPI score heatmap were analyzed and constructed using the STRING database (PPI enrichment *P* value < 1.0E−16). We found that 12 proteins were predicted to have direct connection with β-catenin (Fig. 4c) and 6 proteins among them got the PPI score more than 0.95, including FYN (0.999), GSK-3B (0.998), AKT1 (0.997), GSK-3A (0.985), MAPK9 (0.972), and AKT2 (0.952) (Fig. 4d). The GO enrichment analysis was performed using DAVID (The Database for Annotation, Visualization and Integrated Discovery), including analyzing the molecular function, cellular component, and biological process. The GO analysis of the molecular function (Fig. 4e) and the biological process (Fig. 4f) revealed that NE promoted protein kinase activity, protein phosphatase activity, protein phosphorylation, and intracellular signal transduction in SK-OV-3 cells. Cellular component analysis indicated that NE significantly improved the WNT/β-catenin signaling pathway in SK-OV-3 cells (Fig. 4g). The Reactome pathway database was used to analyze the enhanced biomolecular pathways in NE-treated SK-OV-3 cells. Results showed that the PI3K/AKT signaling pathway was the most significant upregulated signaling pathway in SK-OV-3 cells after being treated with NE. Moreover, the phosphorylation substrates of AKT in both cytosol and nucleus were enhanced by NE treatment (Fig. 4h). NE-affected protein domains were analyzed by the InterPro (Fig. 4i), SMART (Fig. 4j), and Pfam database (Fig. 4k). We found that kinase-associated domains were significantly upregulated by NE. These results suggest that NE endows tremendous influence on the phosphorylation of various phosphor-kinases in SK-OV-3 cells and the AKT signaling pathway probably plays an important role in NE-mediated stabilization of β-catenin.

### NE activates the β-catenin/SLUG axis through phosphorylating AKT in vitro

**Fig. 5:**
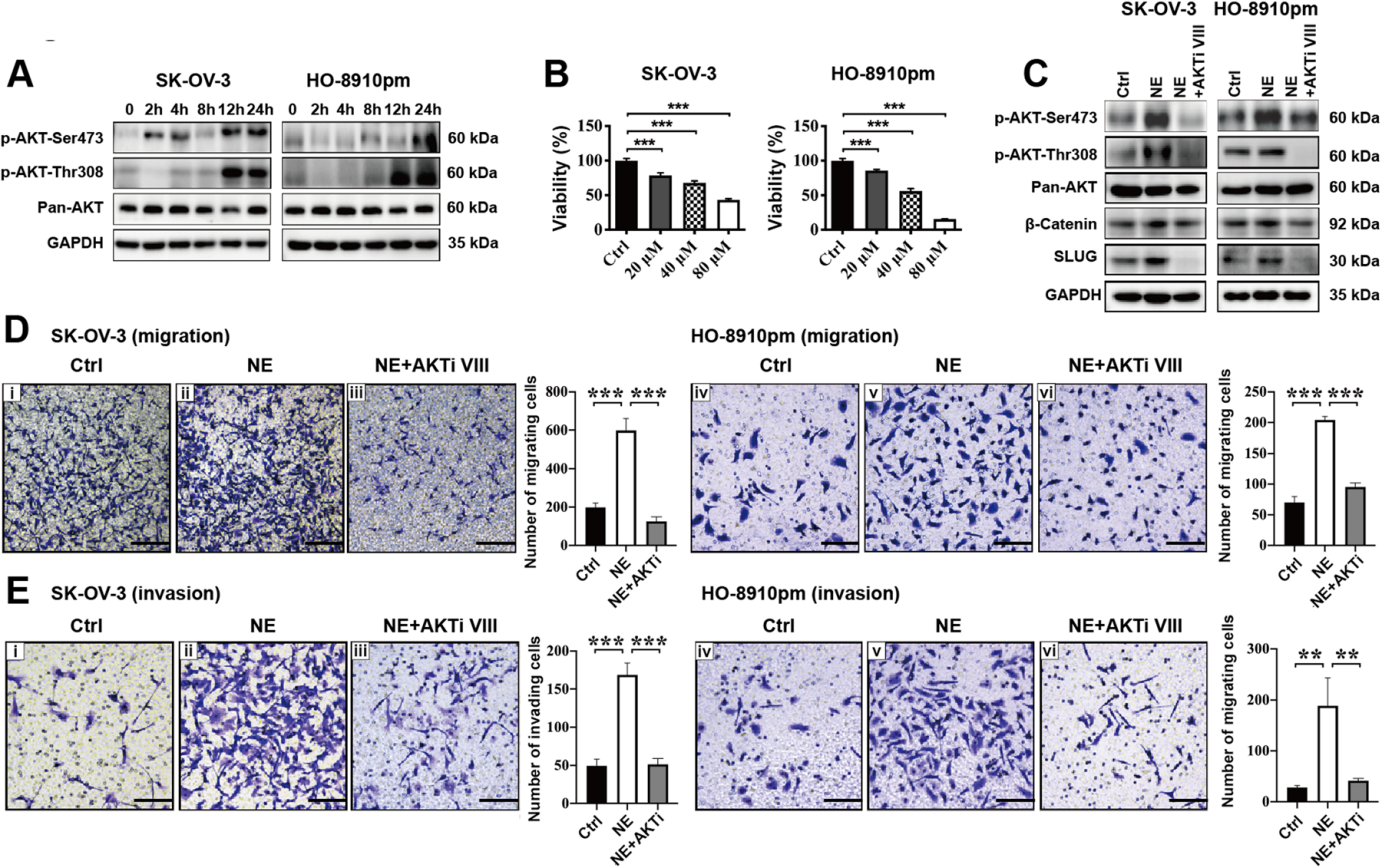
NE activates the β-catenin/SLUG axis through phosphorylating AKT. **a** NE phosphorylates the Ser473 and Thr308 residues of AKT in ovarian cancer cells by using western blotting at 24 h. **b** CCK-8 assay was used to evaluate the effects of different concentration of AKT inhibitor AKTi VIII on the viability of ovarian cancer cells at 24 h. **c** Western blotting showed that AKTi VIII inhibited NE-mediated activation of the AKT/β-catenin/SLUG axis in ovarian cells at 24 h. AKTi VIII reduces NE-mediated pro-migration (**d**) and pro-invasion (**e**) influence on ovarian cancer cells at 24 h. **P < 0.01; ***P < 0.001. Scale bar, 200 μM.

It is reported that AKT promotes invasion of A431 human epidermoid carcinoma cells via increasing the transcriptional activity of β-catenin^27^. Whether AKT participates in NE-mediated activation of the β-catenin/SLUG axis in EOC cells needs to be clarified. First, the results of western blotting showed that NE increased the phosphorylation levels of Ser473 and Thr308 residues in both SK-OV-3 and HO-8910pm cells time-dependently (Fig. 5a). AKT inhibitor (AKTi VIII) was used to downregulate NE-enhanced AKT phosphorylation. We found that AKTi VIII significantly inhibited the viability of EOC cells dose-dependently in vitro (Fig. 5b). Using western blotting, we observed that 10 μM AKTi VIII decreased the phosphorylation levels of both Ser473 and Thr308 residues in EOC cells after being treated with NE (Fig. 5c). Furthermore, AKTi VIII disturbed NE-mediated upregulation of the β-catenin/SLUG axis in EOC cells in vitro using western blotting (Fig. 5c). AKT inhibitor also decreased the migration (Fig. 5d) and invasion (Fig. 5e) of NE-treated EOC cells in vitro. These data indicate that AKT phosphorylation participates in NE-induced activation of the β-catenin/SLUG axis in EOC cells.

### The expression of NE and β-catenin are associated with poor clinical stage in serious ovarian cancer

**Fig. 6:**
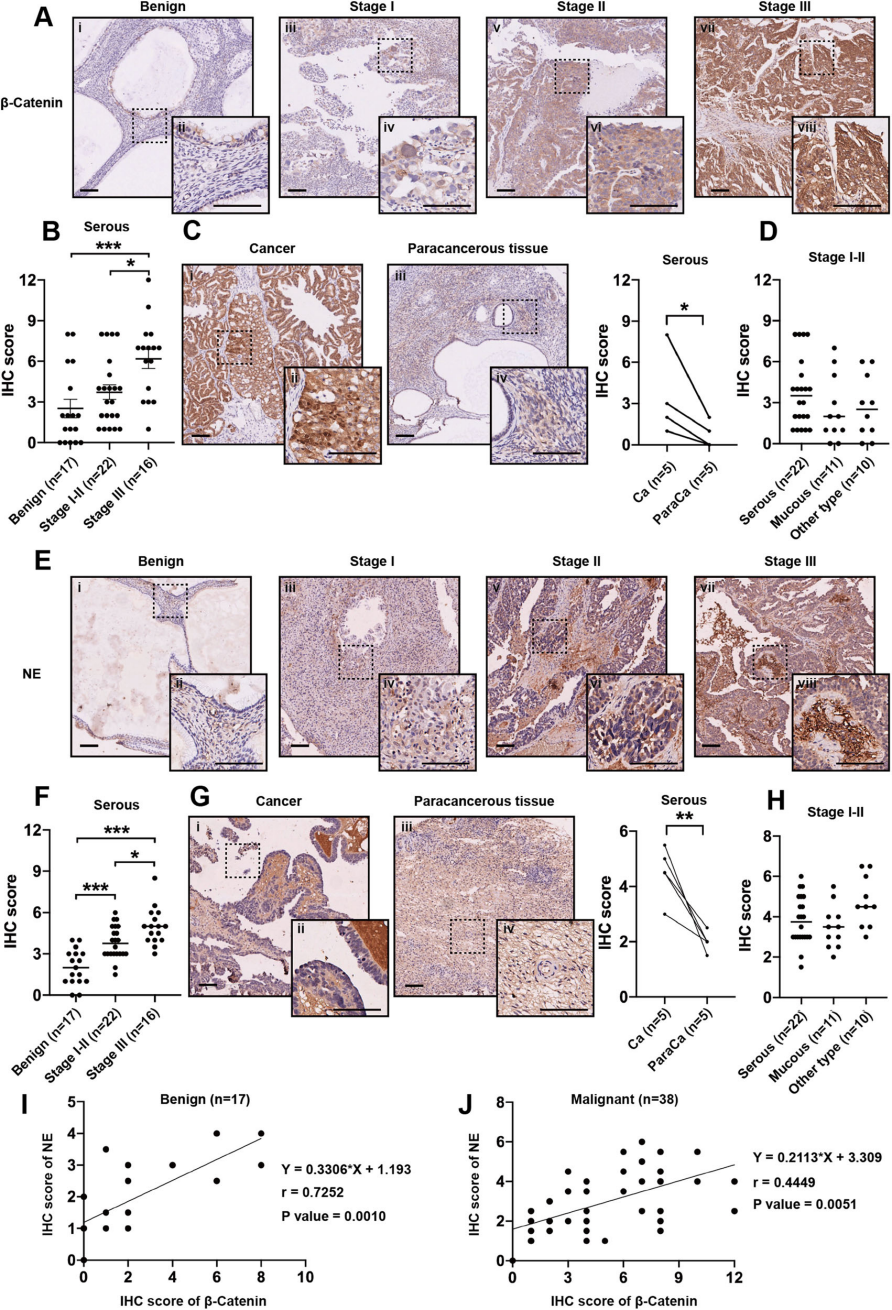
The expression of NE and β-catenin and their clinicopathologic significance in ovarian cancer tissues. Representative images of β-catenin (**a**) and NE (**e**) expression in benign ovarian disease and different FIGO stages of ovarian cancers. The IHC scores of β-catenin (**b**) and NE (**f**) in benign ovarian disease and different FIGO stages of ovarian serous cancer. The IHC scores of β-catenin (**c**) and NE (**g**) in cancerous tissue and paracancerous tissues. The IHC scores of β-catenin (**d**) and NE (**h**) in different pathological types of stages I–II ovarian cancer. The correlation analysis between the β-catenin expression and the NE content in benign ovarian disease (**i**) and ovarian serous cancer (**j**). *P < 0.05; **P < 0.01; ***P < 0.001. Scale bar, 200 μM.

To further investigate the expression of NE and β-catenin in ovarian cancer and analyze their clinicopathological significance, the expression levels of NE and β-catenin were assayed in 17 ovarian cysts, 38 serous carcinomas, 14 mucous carcinomas, and 19 other pathological subtypes of ovarian cancer by tumor tissue arrays (Table 1). We observed that β-catenin was most expressed in cancer cells and rarely in tumor stroma (Fig. 6a). The expression of β-catenin increased with the malignancy of ovarian serous cancer (Fig. 6b). We also found that the expression of β-catenin in cancerous tissues was significantly higher than that in paracancerous tissues (Fig. 6c). However, there was no significance of β-catenin expression among various subtypes of stages I–II ovarian cancer (Fig. 6d). NE was localized in both cancer cells and tumor stroma (Fig. 6e). We observed that the tendency of NE expression in clinical samples was consistent with that of β-catenin, including higher expression in stage II-III ovarian serous cancer (Fig. 6f), higher expression in cancerous tissues (Fig. 6g), and no significant expression in various cancer subtypes (Fig. 6h). We further evaluated the correlation between β-catenin and NE in ovarian benign tumors or ovarian serous cancer separately and the results showed that NE expression was significantly related to β-catenin expression in both benign (Fig. 6i) and malignant (Fig. 6j) ovarian tumors. In summary, these findings suggest that higher expression level of NE and β-catenin might be associated with ovarian cancer progression.

**Table 1:** Clinicopathological characteristics of the 88 patients.

Histological type	FIGO	number	Age (years)	Volume (cm^3^)	NE IHC score	β-catenin IHC score
Ovarian cyst						
Serous	–	8	40.8 ± 15.1	–	2.03 ± 1.28^###^	2.53 ± 2.81^###^
Mucous	–	9				
Ovarian cancer						
Serous	I	4	54.4 ± 11.5	40.7 ± 37.2	3.82 ± 1.19*	3.73 ± 2.49*
	II	18				
	III	16	54.1 ± 8.1	30.04 ± 26.92	5.00 ± 1.33	6.19 ± 2.83
Mucous	I	5	52.1 ± 14.0	51.4 ± 38.0	3.61 ± 1.10	2.50 ± 2.31
	II	7				
	III	2				
Mixed	I	1	47.4 ± 17.3	46.9 ± 42.7	4.58 ± 1.19	2.79 ± 2.70
	II	1				
Endometrioid	II	4				
Clean cell	I	1				
	II	3				
Germ cell	I	4				
MMMT	II	1				
Sex cord-mesenchymal	II	3				
TCC	III	1				

Human ovarian tumor tissue microarrays were used to evaluate the content of NE and the expression of β-catenin in tumor tissues obtained from patients with ovarian cyst and ovarian carcinoma.

*FIGO* International Federation of Gynecology and Obstetrics, *NE* norepinephrine, *IHC score* immunohistochemistry score, *MMMT* ovarian malignant mesoderm mixed tumor, *TCC* ovarian transitional cell carcinoma.

*P < 0.05; ***P or ^###^*P* < 0.001.

^a^ * represents the Comparison of the IHC scores of NE or β-catenin between phase I/II serous ovarian cancer group and phase III serous ovarian cancer group.

^b^^#^ represents the Comparison of the IHC scores of NE or β-catenin between ovarian cyst group and phase I/II serous ovarian cancer group.

### Melatonin inhibits CRS-mediated metastasis-facilitating effects on EOC cells via downregulating the NE/AKT/β-catenin/SLUG axis in nude mouse models and in vitro

**Fig. 7:**
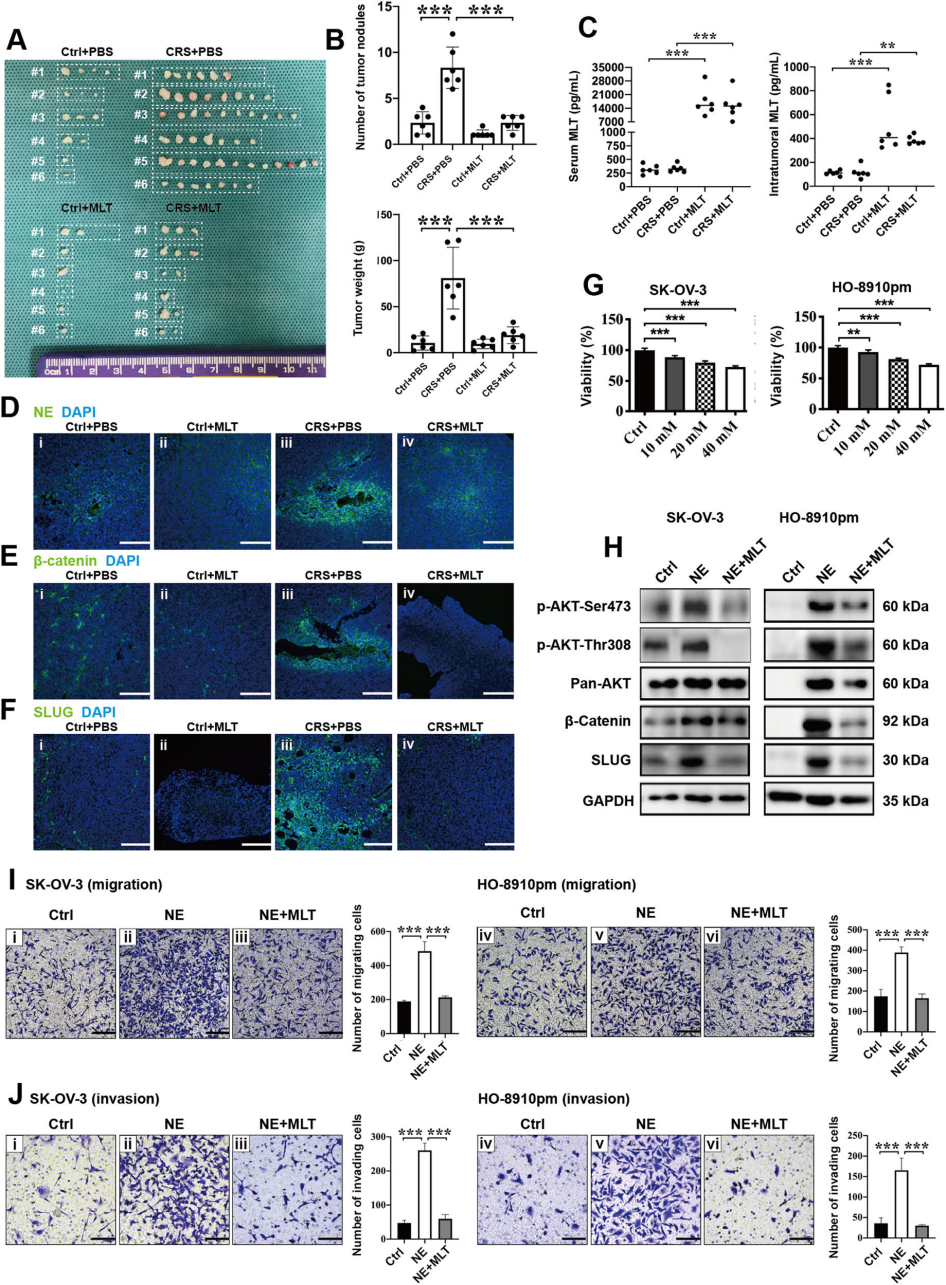
Melatonin inhibits CRS-mediated metastasis-facilitating effects on EOC cells via downregulating the NE/AKT/β-catenin/SLUG axis in nude mouse models and in vitro. **a** Gross morphology of the abdominally implanted tumor burden of ovarian cancer cells (*n* = 6). **b** Quantification of abdominal tumor nodules and tumor weights (*n* = 6). **c** The content of MLT in mouse serum and tumor tissues was evaluated by ELISA (*n* = 6). Representative images of IF staining detecting NE (**d**), β-catenin (**e**), and SLUG (**f**) in tumor tissues. **g** CCK-8 assay was conducted to test the effects of different concentration of MLT on the viability of ovarian cancer cells at 24 h. **h** Western blotting showed that MLT inhibited NE-induced activation of AKT/β-catenin/SLUG axis in ovarian cells at 24 h. MLT reduced the migration (**i**) and invasion (**j**) ability of NE-treated ovarian cancer cells at 24 h. *P < 0.05; **P < 0.01; ***P < 0.001. Scale bar, 200 μM.

MLT is reported to exert antiangiogenesis effects against ovarian cancer through inhibiting AKT phosphorylation in mouse models^28,29^. However, whether MLT exerts anti-metastasis effects against EOC cells in the CRS nude mouse models still remains unknown. First, we established animal models to test the effects of MLT on SK-OV-3 cells in vivo with or without CRS, which were classified into four groups, including Ctrl + PBS, Ctrl + MLT, CRS + PBS, and CRS + MLT (2 × 10^6^ cells per mouse). Mice in the MLT group received daily intraperitoneal injection of MLT (200 µg/100 g per day)^28^. In comparison with the PBS-injected mice, mice received MLT injection from the Ctrl group and the CRS group showed a significant reduction in tumor size (Fig. 7a), weight, and number of abdominal tumor nodules (Fig. 7b). After being intraperitoneally injected with MLT every day, we observed that the concentration of MLT both in serum and tumor tissues were significantly higher than that in mice injected with PBS using ELISA (Fig. 7c). By IF staining, we found that MLT significantly inhibited CRS-induced upregulation of NE (Fig. 7d), β-catenin (Fig. 7e), and SLUG (Fig. 7f) expression in tumor tissues.

CCK-8 assay showed that MLT inhibited the viability of EOC cells in vitro (Fig. 7g) and we chose 10 μM to perform the subsequent experiments. Western blotting showed that MLT reduced NE-induced upregulation of AKT phosphorylation, β-catenin expression, and SLUG expression in EOC cells in vitro (Fig. 7h). MLT decreased the migration (Fig. 7i) and invasion (Fig. 7j) ability of NE-treated EOC cells in vitro. In addition, we conducted the wound-healing assays (Supplementary Fig. 1) and the 3D tumor spheroid invasion assays (Supplementary Fig. 2) to evaluate the effects of NE/AKT/β-catenin signaling transduction and MLT on the motility of EOC cells in vitro at 48 h, which were consistent with the results of transwell assays. These results showed that MLT exerted inhibitory effects on CRS or NE-mediated pro-metastatic effects on ovarian cancer cells in nude mice and in vitro through downregulating the NE/AKT/β-catenin/SLUG axis.

### Melatonin suppresses β-catenin-mediated transcriptional regulation of SLUG expression in EOC cells

**Fig. 8:**
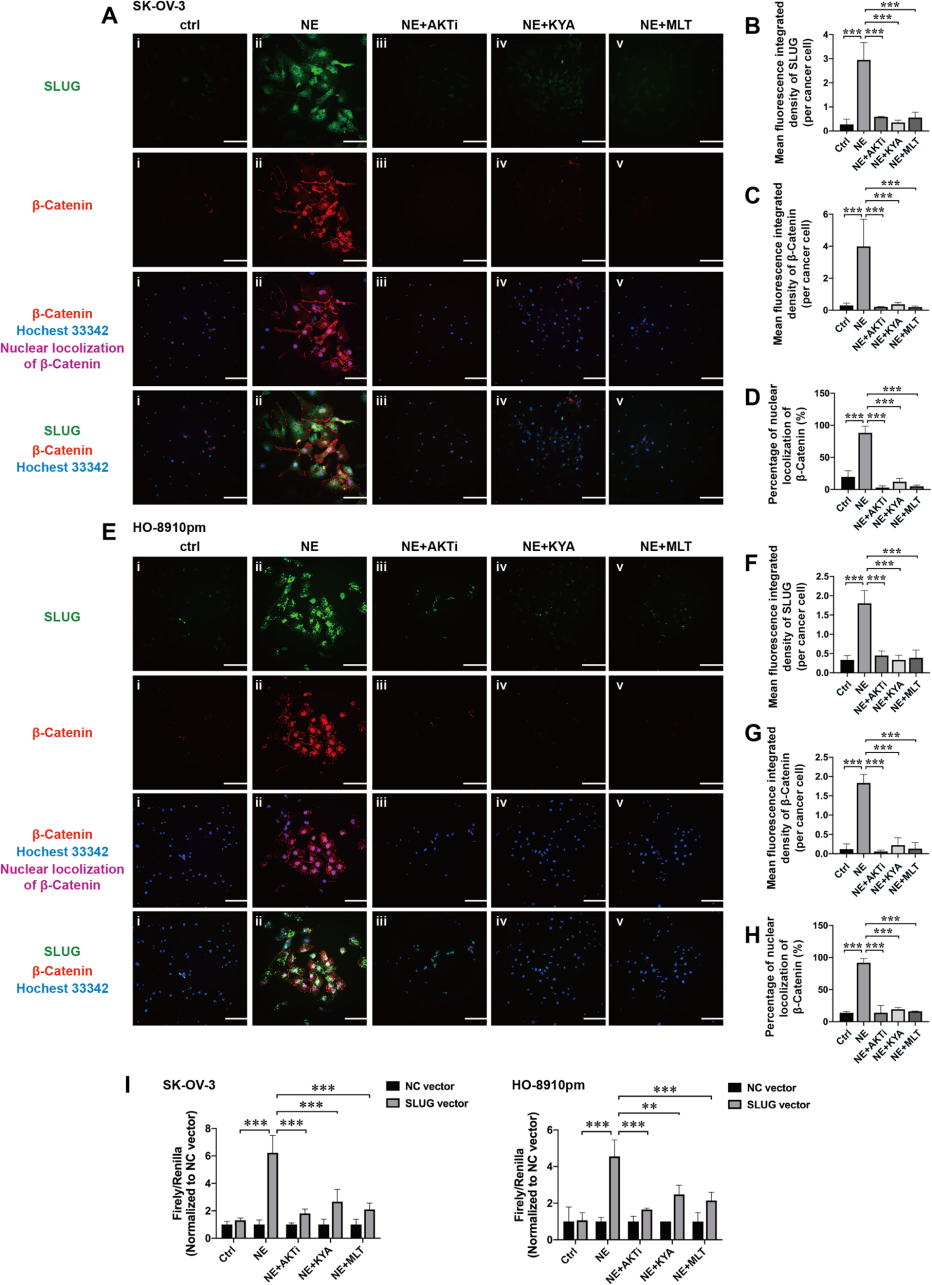
Melatonin suppresses β-catenin-mediated transcriptional regulation of SLUG expression in EOC cells. Representative images of IF staining detecting the effects of NE, AKTi, KYA and melatonin on the SLUG expression (green), β-catenin expression (red), and nuclear localization of β-catenin (purple) in SK-OV-3 cells (**a**) and HO-8910pm cells (**e**) at 24 h. Quantification of the mean fluorescence integrated densities of SLUG (**b**, **f**) and β-catenin (**c**, **g**) in EOC cells (*n* = 3). The percentage of nuclear localization of β-catenin in SK-OV-3 cells (**d**) and HO-8910pm cells (**h**) (*n* = 3). **i** Dual luciferase reporter assays with the SLUG’s promoter reporter plasmid (pGL3-basic vector) were used to evaluate the effects of NE, AKTi, KYA and melatonin on the activity of SLUG’s promoter in EOC cells at 24 h (*n* = 3). **P < 0.01; ***P < 0.001. Scale bar, 200 μM.

It is well-known that β-catenin interacts with T-cell factor 4 in the WNT signaling transduction and exerts transcriptional regulation of EMT-related target genes, including SLUG^30^. Here, we conducted double staining IF and luciferase assay to evaluate whether β-catenin transcriptionally regulate SLUG expression in NE-treated EOC cells, which has never been reported before. Results from IF showed that NE significantly increased the expressions of β-catenin and SLUG in both of SK-OV-3 and HO-8910pm cells which were downregulated by AKT inhibitor, β-catenin inhibitor, and MLT (Fig. 8a, e). We calculated the mean fluorescence integrated densities of SLUG (green fluorescence, Fig. 8b, f) and β-catenin (red fluorescence, Fig. 8c, g), respectively, which showed that NE significantly increased their expressions in EOC cells. In addition, NE promoted the nuclear localization of β-catenin (purple fluorescence, Fig. 8d, h) in EOC cells, which indicated the transcriptional regulation of β-catenin in EOC cells. Furthermore, we used dual luciferase assay with SLUG’s promoter reporter vector to evaluate the β-catenin-mediated transcriptional regulation of SLUG in EOC cells. Results showed that NE significantly promoted the activation of SLUG’s promoter in EOC cells, which was reduced by AKTi, KYA, and MLT (Fig. 8i). Combined with the data from western blotting and IF staining, these results suggest that NE promotes SLUG expression through improving nuclear localization and transcriptional function of β-catenin in EOC cells.

## Discussion

Our results demonstrate that CRS promoted the abdominal metastasis of EOC, and activated the expression of EMT-related molecules. Moreover, CRS-induced NE promoted the migration and invasion of ovarian cancer cells and increased the activity of SLUG promoter through promoting expression and nuclear localization of β-catenin, which is regulated by phosphorylating AKT. Moreover, the expression levels of both NE and β-catenin were confirmed in tumor tissues and their expression levels were associated with poor clinical stage of serous EOC. In addition, MLT effectively reduced the abdominal tumor burden of ovarian cancer induced by CRS through inhibiting the NE/AKT/β-catenin/SLUG axis in nude mouse models and in vitro, suggesting a potential therapeutic relevance.

There are various methods to evaluate the impacts of chronic stress, including psychological scales, measuring serologic indexes, conducting behavioral experiments, etc. Which one or which combination is better still unclear. Susan et al. reported the significant relationships between stress-related IL-6, cortisol, and vegetative depression (measured by The Center for Epidemiological Studies-Depression Scale) in patients with ovarian cancer^31^. However, Elizabeth et al. found no significant association between ovarian cancer risk and depression symptoms (measure by The Crown-Crisp phobic anxiety index)^32^. The two controversial findings demonstrated that it was difficult to overcome confounding factors and fully characterize an individual’s subjective experience of stress via several psychological scales and symptoms. The endogenous NE is released from adrenal medulla and sympathetic nerves during chronic stress. Whether it is reasonable to use circulating NE as a prognostic indication in cancer patients needs to be further studied. Although previous studies suggest the pro-carcinogenic effects of NE, it seems that the content of intratumoral NE might be a better medical indication. By using a denervation strategy to decrease circulating Epi, Adam et al. found that circulating Epi and NE were dispensable for the effects of chronic stress on breast cancer metastasis and suggested a more important role played by NE released from the sympathetic nerve terminals in tumor microenvironment^33^. Jennifer et al. found that the level of intratumoral NE was positively related to the activity of superoxide dismutase 2^34^, which contributed to the transcoelomic metastasis in ovarian cancer^35^. Julie et al. reported that the intratumoral NE exerted neurotrophic effect through BDNF induction, whose high expression correlated with poor outcome in ovarian cancer patients^10^. In this study, we first found that intratumoral NE was positively correlated with clinical stage in patients with ovarian serous cancer. These data suggest that the important role of the sustained pro-metastatic neuro-endocrine regulatory network in tumor microenvironment and the potential application of NE as a novel prognostic marker in ovarian cancer.

The stabilization of β-catenin is critical in the process of cancer metastasis, which is usually determined by the balance between the activities of phosphorylation and dephosphorylation regulated by various kinases and phosphatases. In this study, we found that NE significantly increased the phosphorylation of various kinase’s residues, including AKT-Ser473/Thr308, GSK-3α/β-Ser9/21, p27-Thr198, p70S6K-Thr389, PRAS40-Thr246, etc. Furthermore, we found that NE-induced β-catenin expression and migration/invasion of ovarian cancer cells could be reversed by the AKT inhibitor in vitro. Fang et al. observed that AKT phosphorylated β-catenin at Ser552 residue, increased its transcriptional activity and promoted cancer invasion and development^27^. AKT kinase also phosphorylated GSK-3β and inhibited its ability to phosphorylate, which resulted in the degradation of β-catenin^36^. Besides the AKT/GSK-3/β-catenin axis, AKT kinase is reported to promote cancer metastasis through phosphorylating p27-Thr198, p70S6K-Thr389, and PRAS40-Thr246 residues. Zhao et al.^37^ found an oncogenic feedforward loop between the p27-Thr198/c-Jun N-terminal kinase2/Signal Transducers and Activators of Transcription3/TWIST axis and AKT, whose activation promoted EMT and cancer metastasis. Franziska et al. reported that the pan-AKT inhibitor GDC-0068 inhibited the phosphorylation level of p70S6K-Thr389 and PRAS40-Thr246 residues and reduced the brain metastasis of PIK3CA-mutant breast cancer^38^. These data demonstrated the AKT-mediated pro-cancerous cascades and the potential downstream targets of it in ovarian cancer. However, how NE activates AKT kinase and which residues within β-catenin react to AKT need to be further discovered in mouse models and in vitro which might contribute to drug discovery in ovarian cancer treatment.

Intriguingly, we found that MLT inhibited the abdominal tumor burden of ovarian cancer induced by CRS in nude mouse models, which might be associated with MLT-mediated inhibition of the NE/AKT/β-catenin/SLUG axis. MLT has been shown extensive anticancer effects with no severe effects which makes it a potential and promising agent in ovarian cancer therapy^13,39^. Some findings support our observation that AKT and β-catenin are targets of MLT. Mao et al.^40^ found that MLT inhibited EMT and migration in breast cancer through disturbing the AKT-Ser473/GSK-3β-Ser9/β-catenin/SNAIL axis. Liu et al.^41^ showed that MLT inhibited β‐catenin activation and stemness in chordoma through decreasing the phosphorylation level of Y86/Y333/Y654 residues in β-catenin. Chao et al.^42^ found that MLT decreased EMT and metastasis in lung cancer through targeting the β-catenin/TWIST axis. Compared with various clinical evaluation of MLT in patients with non-small-cell lung cancer, gastrointestinal tract cancer, or breast cancer (systematically reviewed in^43^), effects of clinical application of MLT on patients with ovarian cancer are limited. Lissoni et al.^44^ reported a phase II study of tamoxifen plus MLT in untreatable metastatic solid tumor patients, including two patients with ovarian cancer. The first person had lung metastasis, achieved no response to MLT, and lived for 4 months. Second person had both lung and nodes metastasis, achieved stabilized disease, and lived for 10 months. Although MLT exerts significant tumor-inhibitory effects on ovarian cancer in vitro and in animal models, more clinical studies are needed to draw more definite conclusions in ovarian cancer therapy.

Collectively, we first reported a new molecular mechanism that NE-mediated activation of the AKT/β-catenin/SLUG axis promoted ovarian cancer metastasis induced by CRS, which partially suggested the necessity of psychological, behavioral, pharmacological intervention against negative emotions in patients with ovarian cancer. We also found that MLT inhibited NE-induced pro-metastatic effects on ovarian, which supported the potential clinical application of MLT in ovarian cancer treatment. Further studies will be focused on the direct regulation of how NE activates AKT kinase and which phosphorylation residue within β-catenin reacts to the NE/AKT signaling pathway. Moreover, whether it is beneficial for chemotherapy combined with MLT in ovarian cancer patients need to be further investigated in the future clinical trial.

## Materials and methods

### Cell lines culture

SK-OV-3 and HO-8910pm were bought from Shanghai Cell Bank of Chinese Academy of Sciences. Cancer cells are cultured in DMEM/high glucose medium (Thermo Fisher Scientific, USA, AE28846270) with 10% fatal bovine serum (FBS, Bioind, Israel, #04-001-1ACS), and streptomycin/penicillin (100 U/ml, YEASEN, China, #60162ES76). All cell lines were mycoplasma free.

### Establishing chronic restraint stress tumor-bearing mouse models

Six-week-old female BALB/c nude mice were obtained from Shanghai Jiao Tong University (School of Medicine, Shanghai, China). Animal experiments were approved by the Ethics Committee in accordance with the guidelines set by the Institutional Animal Care and Use Committee.

Nude mice were stochastically classified into the control groups and the CRS groups. The method for establishing CRS mouse models was reported previously^17^. The CRS group mice received daily restraint stress for 2 h and mice in control group were placed in cages without food and water at the same time. After being treated with or without pre-CRS for 7 days, SK-OV-3 cells were intraperitoneally injected into nude mice (2 × 10^6^, *n* = 6). Animals were euthanized after anesthesia by inhaling isoflurane (RWD Life Science Co., Ltd, #R510-22) after 35 days. Blood samples were collected, and tumor weight and numbers of abdominal tumor nodules were measured. To assess whether MLT exert anticancer effects against EOC cells in stressed tumor-bearing nude mice, mice in control or CRS group were intraperitoneally injected with PBS or MLT (200 µg/100 g per day; MCE, USA, HY-B0075)^28^ daily at first and last for 35 days. During the experiments, all investigators were not blind to the group allocation. The experiments were replicated four times.

### Enzyme-linked immunosorbent assay

The concentration of corticosterone (IBL, Germany, #RE52211), NE (IBL, #RE59261), and MLT (IBL, #RE54021) in mouse serum and tumor tissues was measured by ELISA (*n* = 6). The blood samples were collected from eyeball, stranded at room temperature (RT) for 2 h, and centrifuged to separate serum (12,000 rpm at 4 °C for 15 min). Tumor tissues were lysed in RIPA (Yeasen, #20101ES60).

### Immunofluorescence (IF) staining and H&E staining

Tumors were fixed by 4% paraformaldehyde (PFA), dehydrated by 30% sucrose, embedded in OCT (Sakura Finetck, USA, #4583), sectioned into 5 µm by cryotome, and stored at −80 °C.

For IF on tumor tissues, frozen tissue sections were air-dried at RT for 1 h, fixed by 4% PFA for 15 min, penetrated by TBS containing 0.025% Triton X-100 at RT for 10 min, blocked with TBS containing 10% FBS + 1% bovine serum albumin (YEASEN, #36101ES25) + 0.3 M glycine + 0.1% Tween 20 for 10 min for 2 h, and incubated with antibodies at 4 °C overnight. The dilutions of antibodies are as followings: TWIST (Ms, 1:100; Abcam, England, ab50887), SLUG (Ms, 1:100; Abcam, ab51772), SNAIL (Rb, 1:100; Abcam, ab216347), β-catenin (Rb, 1:200; Abcam, ab32572), β-catenin (Ms, 1:200; Abcam, ab22656), and NE (Rb, 1:100; Abcam, ab8887). Next day, tissues were stained with fluorescent secondary antibodies at RT for 1 h: Alexa Fluor-488 (Rb, 1:200; Thermo Fisher Scientific, A11008) or −488 (Ms, 1:200; Thermo Fisher Scientific, A11029) or −594 (Rb, 1:200; Abcam, ab150080). Then tissues were counterstained with DAPI (Abcam, ab104139) and photographed by fluorescence microscope or confocal laser scanning microscope (Leica, Germany).

Protocol for IF on cultured cells is reported previously^45^. Antibodies used are as following: SLUG (Ms, 1:100; Abcam), β-catenin (Rb, 1:250; Abcam), Alexa Fluor-488 (Ms, 1:200; Thermo Fisher Scientific), −594 (Rb, 1:200; Abcam), and Hochest 33342 (1 µg/mL, CST, #4082). The fluorescence integrated density was measured by ImageJ (NIH). The experiments were replicated three times.

### Cell Counting Kit-8 (CCK-8) assay

The work concentration of SKL2001 (MCE, HY-101085), NE (MCE, HY-13715), KYA1797K (MCE, HY-101090), AKTi VIII (MCE, HY-10355), and MLT (MCE) was determined by CCK-8 assay. In all, 5000 cancer cells were seeded in 96-well plate and reagents with different concentrations were used to treat cancer cells for 24 h (*n* = 3). Absorbance at 450 nm was measured using Synergy microplate reader (BioTek Instruments, Inc., USA). The experiments were replicated three times.

### Western blotting assay

Cells were lysed in RIPA and protein concentration was quantified by BCA Protein Assay Kit (Thermo Fisher Scientific, NCI3225CH). Protocol for western blotting is reported previously^45^. Antibodies used are as following: β-catenin (Rb, 1:2000; Abcam), SLUG (Ms,1:1000; Abcam), phosphor-AKT-Ser473 (Rb, 1:1,000; CST, 4060), phosphor-AKT-Thr308 (Rb, 1:1,000; CST, 13038), pan-AKT (Rb, 1:1,000; CST, 4691), GAPDH-HRP (Rb, 1:5,000; Yeasen, 30203ES10), horseradish peroxidase‑conjugated anti‑rabbit IgG (1:2,500; Yeasen, #33101ES60), and anti‑mouse IgG (1:2,500; Yeasen, #33201ES60). Electro‑chemiluminescence method was used to visualize blots (New Cell & Molecular Biotech Co., China, #P10100). GAPDH was used as internal standards. The experiments were replicated three times.

### Transwell migration and invasion assay

For invasion assay, 50 μl Matrigel (1:6 dilution, Corning, #356234) was put in chamber with 8 μm pore size (Corning, USA, #3422). Overall, 6 × 10^4^ cells in 150 μl medium were placed in chamber and 0.5 mL medium containing 20% FBS was added to the lower chamber (*n* = 3). After 24 h, the chambers were washed, fixed in 4% PFA for 20 min, stained with 0.25% crystal violet for 30 min, observed, and photographed. Three fields (20×) were selected randomly and the number of cancer cells was counted. For migration assay, the protocol used was the same as for invasion, but without Matrigel. The experiments were replicated three times.

### Human Phospho-Kinase Array Kit and bioinformatic analysis

We used a Human Phospho-Kinase Array Kit (R&D Systems, UK, #ARY003B) to evaluate the effects of NE on phosphorylation of kinases in SK-OV-3 cells. Array was purchased, performed, and analyzed by Univ-bio (Shanghai, China). STRING (https://string-db.org) was used to perform the PPI analysis, GO analysis, pathway analysis, and protein domain analysis.

### Human ovarian tumor tissue microarray

Human ovarian tumor tissue microarrays (#OVD481 and #HovaC07OPT01) were used to evaluate expression of NE (Rb, 1:100; Abcam) and β-catenin (Rb, 1:800; Abcam) in patients, which were purchased from and performed by Shanghai Zuocheng Biotech (Shanghai, China). The calculation of IHC scores is reported previously^46^.

### Wound-healing assay

Overall, 5 × 10^5^ cancer cells per well were seeded in six‑well plates (*n* = 3). After a straight line was scratched, suspending cells were removed by PBS and then 10 μM NE, 10 μM AKTi, 25 μM KYA, and 10 μM MLT were added to the culture medium, respectively, for 48 h. The experiments were replicated three times.

### 3D tumor spheroid invasion assay

Tumor spheroid cells were cultured in growth medium contained with DMEM/F12 (Hyclone, #AC10932967) medium supplemented with 10% KnockOut^TM^ Serum Replacement (Thermo Fisher Scientific, #10828028), 1X non‑essential amino acids (Solaribio, China, #N1250-100), 1 mM sodium pyruvate (Hyclone, #AZH193209), 20 ng/ml human recombinant epidermal growth factor (Thermo Fisher Scientific, #RP-8661), and 10 ng/ml basic fibroblast growth factor (Thermo Fisher Scientific, #RP-8626) for 48 h^45^. Next, tumor spheroids were cultured in growth medium added with Matrigel (1:4 dilution, *n* = 3), different inhibitors, and MLT for 48 h. The diameters of tumor spheroids were measured^47^. The experiments were replicated three times.

### Dual luciferase reporter assay

The SLUG dual luciferase reporter vector (pGL3-basic vector) was constructed by RIBOBIO (Guangzhou, China). Overall, 5 × 10^4^ cancer cells per well were seeded in 24‑well plates. Cells were transfected with 500 ng NC or SLUG reporter vectors by Lipofectamine 3000 (Thermo Fisher Scientific, #L3000015; *n* = 3). Forty-eight hours after transfection, NE, NE + AKTi, NE + KYA, and MLT were added into the culture medium for 24 h. Cells were lysed and measured by Promega Dual-Luciferase Reporter Assay Systems (German, #TM040). The experiments were replicated three times.

### Statistical analysis

Results are shown as the mean ± standard error. The variance is similar between the groups that are being statistically compared. Student’s *t*-test or one-way ANOVA or correlation analysis was used to evaluate statistical differences via GraphPad Prism version 8 (GraphPad Software, Inc., USA). *P* < 0.05 was considered to indicate a statistically significant difference.

## Supplementary information

Supplemental Figure Legends

Supplementary figure 1

Supplementary figure 2
