# Correction: Melatonin suppresses chronic restraint stress-mediated metastasis of epithelial ovarian cancer via NE/AKT/β-catenin/SLUG axis

**DOI:** 10.1038/s41419-020-02958-0

**Published:** 2020-09-08

**Authors:** Shixia Bu, Qian Wang, Junyan Sun, Xiao Li, Tingting Gu, Dongmei Lai

**Affiliations:** 1 The International Peace Maternity and Child Health Hospital, School of Medicine, Shanghai Jiaotong University, Shanghai, 200030 China; 2 Shanghai Key Laboratory of Embryo Original Disease, School of Medicine, Shanghai Jiaotong University, Shanghai, 200030 China; 3 Shanghai Municipal Key Clinical Speciality, Shanghai, 20030 China; 4 Department of Obstetrics and Gynaecology, Shanghai Sixth People’s Hospital, Shanghai Jiaotong University, Shanghai, 200233 China

**Keywords:** Ovarian cancer, Pharmaceutics

Correction to: *Cell Death and Disease*

10.1038/s41419-020-02906-y published online 18 August 2020

The original version of this article contained errors in the author affiliations.

Author Xiao Li was incorrectly associated with Department of Obstetrics and Gynaecology, Shanghai Sixth People’s Hospital, Shanghai Jiaotong University, Shanghai 200233, China. The correct affiliation is Shanghai Municipal Key Clinical Speciality, Shanghai 20030, China.

This has now been corrected in both the PDF and HTML versions of the article.

