# Supplementary material for: Melatonin suppresses chronic restraint stress-mediated metastasis of epithelial ovarian cancer via NE/AKT/β-catenin/SLUG axis
Source: Cell Death Dis. 2020 Aug 18;11(8):644. doi: 10.1038/s41419-020-02906-y (PMC7435194; doi:10.1038/s41419-020-02906-y)
Supplement: Supplementary file 1 — Supplemental Figure Legends [file 41419_2020_2906_MOESM1_ESM.docx]

**Supplemental Figure 1. Melatonin reduces the NE-induced facilitating wound-healing ability of EOC cells in vitro.** The wound-healing assays were conducted to further evaluate the effects of NE/AKT/β-catenin signaling transduction and melatonin on the migration ability of SK-OV-3 cells (**a**) and HO-8910pm cells (**b**) in vitro at 48 h. ***P, <0.001. Scale bar, 200 μM.

**Supplemental Figure 2. Melatonin reduces the NE-induced facilitating invading ability of EOC cells in vitro by 3D invasion assay.** The 3D tumor spheroid invasion assays were conducted to further evaluate the effects of NE/AKT/β-catenin signaling transduction and melatonin on the invasion ability of SK-OV-3 cells (**a**) and HO-8910pm cells (**b**) in vitro at 48 h. ***P, <0.001. Scale bar, 200 μM.
