# Supplementary figures and images for: Melatonin suppresses chronic restraint stress-mediated metastasis of epithelial ovarian cancer via NE/AKT/β-catenin/SLUG axis
Source: Cell Death Dis. 2020 Aug 18;11(8):644. doi: 10.1038/s41419-020-02906-y (PMC7435194; doi:10.1038/s41419-020-02906-y)

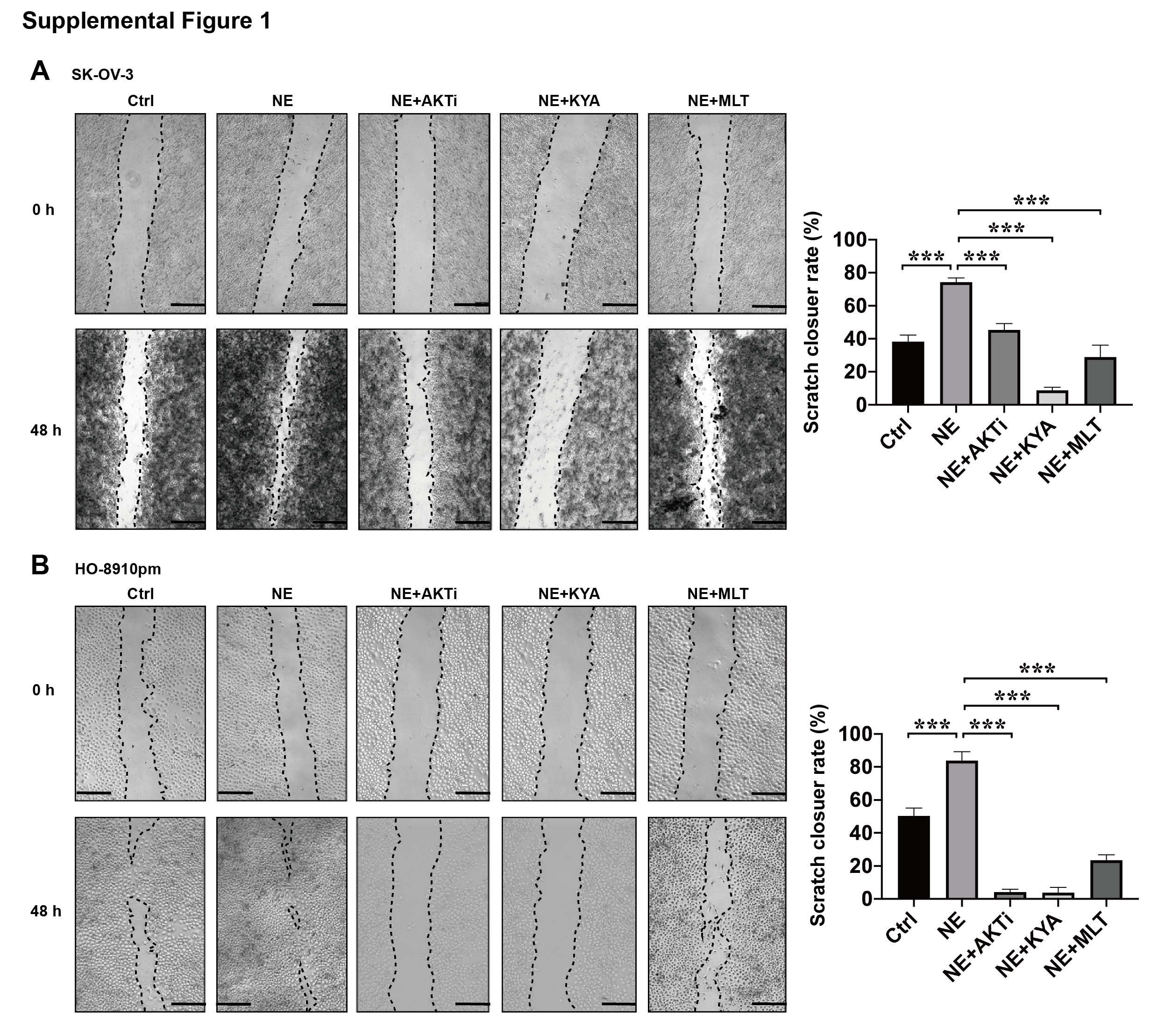

Supplement: Supplementary file 2 — Supplementary figure 1 [file 41419_2020_2906_MOESM2_ESM.png]

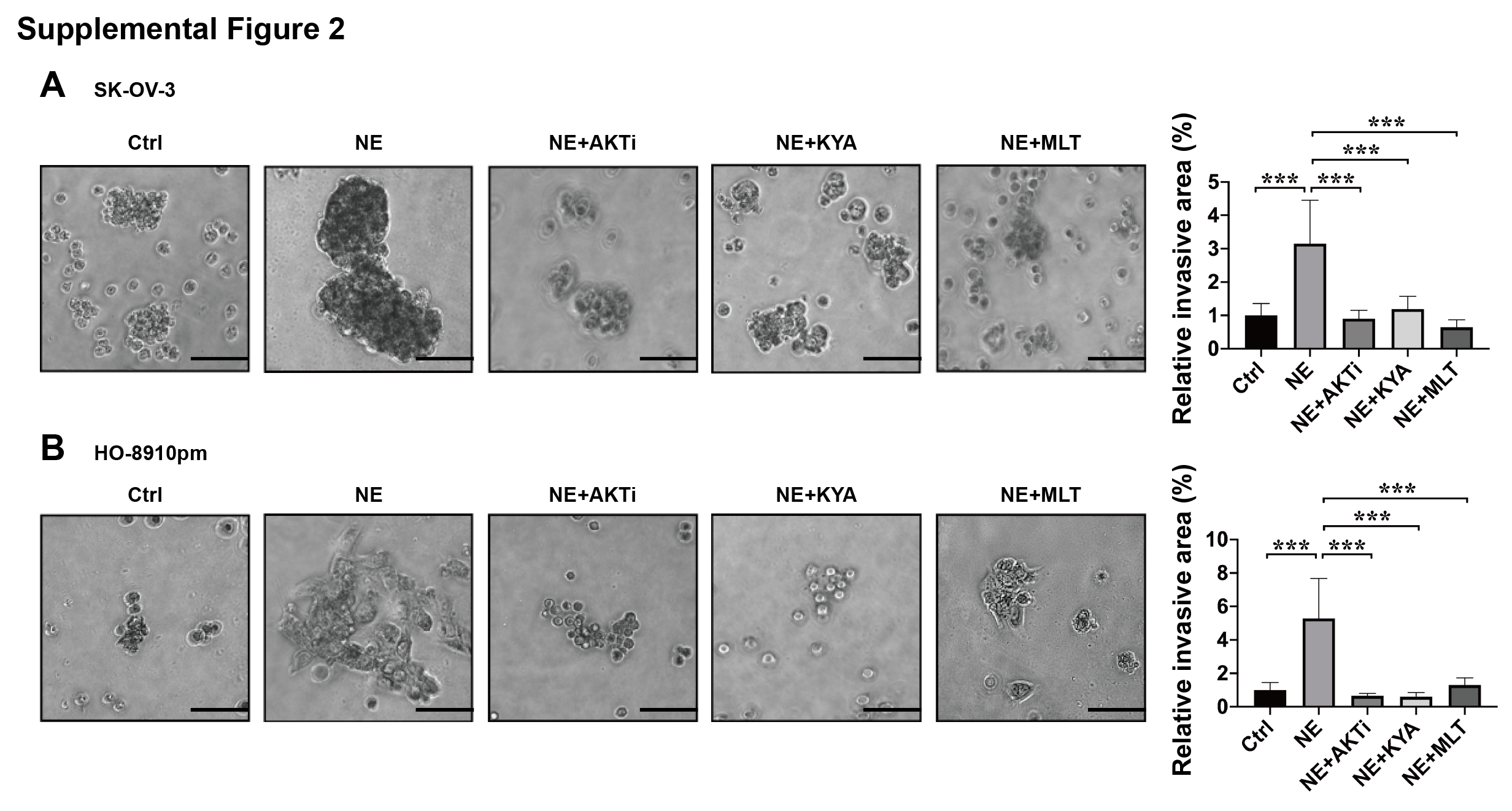

Supplement: Supplementary file 3 — Supplementary figure 2 [file 41419_2020_2906_MOESM3_ESM.png]
